# Peripherally inserted central catheter related pericardial effusion/cardiac tamponade in neonates: Analysis of two cases and literature review

**DOI:** 10.1097/MD.0000000000035779

**Published:** 2023-10-27

**Authors:** Yucen Liu, Maojun Li, Wei Shi, Binzhi Tang

**Affiliations:** a School of Medicine, University of Electronic Science and Technology, Chengdu, China; b Department of Pediatrics, Sichuan Academy of Medical Sciences and Sichuan Provincial People’s Hospital, Chengdu, Sichuan Province, China.

**Keywords:** cardiac tamponade, neonates, pericardial effusion, peripherally inserted central catheter

## Abstract

**Rationale:**

Peripherally inserted central catheter (PICC)-related pericardial effusion/cardiac tamponade is a rare but fatal complication which cause a high mortality if not timely diagnosed and treated. However, the atypical manifestations and the rapid deterioration present challenges for neonatologists, and there has been limited investigation reported globally to date. Furthermore, a systematic review and comprehensive summary of clinical management are lacking. The significance of this article lies in emphasizing the importance of maintaining vigilance in high-risk neonates and implementing effective management strategies for PICC-related pericardial effusion/cardiac tamponade, thereby contributing to saving more lives.

**Patient concerns:**

In the current report, we discuss 2 cases of neonatal pericardial effusion/cardiac tamponade following PICC catheterization.

**Diagnosis:**

The first case was diagnosed based on forensic autopsy and the second case was diagnosed by bedside echocardiography.

**Interventions and outcomes:**

The first case was treated conservatively and the second case underwent pericardiocentesis, unfortunately both were died.

**Lessons:**

Once sudden hemodynamic or respiratory abnormalities are detected in neonates with PICC placement, particularly in the preterm infants, prompt diagnosis by cardiac ultrasound is required to verify pericardial effusion/cardiac tamponade and immediate pericardiocentesis or pericardiotomy is necessary to improve survival.

## 1. Introduction

Peripherally inserted central catheter (PICC) provides ideal venous access for critically ill neonates, and has been widely utilized in neonatal intensive care unit (NICU).^[1,2]^ However, a series of PICC-related complications have been reported, including thrombosis/occlusion, catheter-related infection, rupture of catheter, and malposition of the catheter.^[2–5]^ Pericardial effusion/cardiac tamponade is a rare but fatal complication,^[4]^ with reported incidence rates ranging from 0.07% to 2%^[6]^ and a mortality rate as high as 50%.^[7]^ In the past ten years, approximately 1500 neonates underwent PICC catheterization in our NICU. Among these, 2 cases of PICC-related pericardial effusion/cardiac tamponade occurred, with an incidence rate of about 0.13%, which was consistent with previous studies.^[6]^ This study is aimed to improve the prevention and treatment of neonatal pericardial effusion/cardiac tamponade related to PICC by retrospectively analyzing and summarizing clinical characteristics of the 2 cases, and reviewing relevant literature published in recent years.

## 2. Case presentation

### 2.1. Case 1

**Figure 1.**
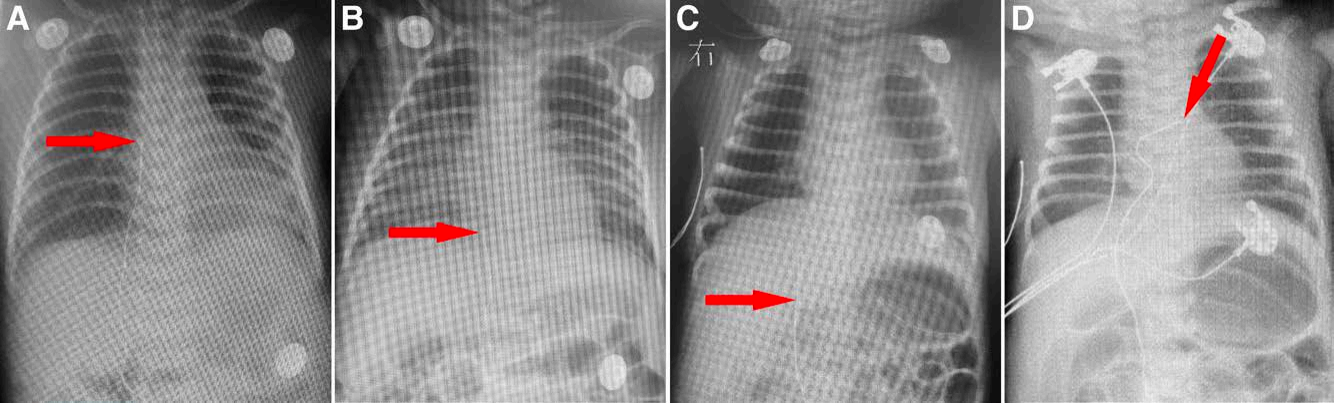
Positioning of catheter tip by chest radiography following peripherally inserted central catheter (PICC) placement, the locations of catheter tip were indicated by the red arrows. (A) PICC tip was positioned at the level of the pulmonary artery, approximately 30 mm from thoracic vertebra (T) 9 at postnatal day (PND) 2 in case 1; (B) PICC tip was located to the right side of T 9 at PND 6 in case 1; (C) PICC tip was positioned at the level of lumbar vertebra (L) 1 at PND 2 in case 2; (D) the PICC catheter traversed the vertebral column at the right side of T 6 and terminated to the left of T 5 at PND 3 in case 2.

A male neonate, with birth weight (BW) of 1780 g, was delivered vaginally at gestational age (GA) of 32^6/7^ weeks due to maternal premature rupture of membranes (PROM) for 6 hours. He appeared dyspnea and hypotonia, and pulmonary infection was revealed by chest x-ray (CXR) and blood examination (white blood cells [WBC] 4.91 × 10^9^/L, neutrophil [NEU] 1.6 × 10^9^/L; procalcitonin 27.98 ng/mL). Invasive mechanical ventilation (MV) was used from postnatal day (PND) 1 to PND 7, followed by transition to noninvasive MV until PND 11. Infection was effectively controlled by antibiotics treatment as evidenced by the results of blood reexamination (WBC 7.23 × 109/L, NEU 4.01 × 109/L; procalcitonin 0.3 ng/mL). Patent foramen ovale and tiny patent ductus arteriosus (PDA) were found by echocardiography at PND 10. PICC was inserted through the left great saphenous vein at PND 2, and then parenteral nutrition (PN) was administrated via PICC every day. PICC tip positioning by postinsertion CXR were shown in Figure 1A and B. At PND 13 or 11 days after PICC placement, saturation of pulse oxygen (SpO_2_) suddenly declined to 35% and heart rate (HR) dropped to 105 beats/min, accompanied by weak spontaneous respiration and cyanotic appearance. Airway suctioning was immediately performed and some milk was drained, then oxygen inhalation was provided via facial mask connecting to a T-piece resuscitator. The baby was promptly intubated in response to the progressive decline of SpO_2_ and HR, with copious amounts of milky substance emanating from both the oral cavity and glottis. Following further airway suctioning, positive-pressure ventilation via a T-piece resuscitator was initiated along with chest compressions, followed by intravenous administration of saline boluses, epinephrine and sodium bicarbonate; however, neither SpO_2_ and HR showed any signs of improvement. No pericardiocentesis was performed in the hospital, and forensic autopsy excluded gastro-esophageal reflux-induced asphyxia as the cause of death, as microscopic examination revealed abundant chylous effusion (fat globule ++) in the pericardium, indicating that chylo-pericardial effusion induced cardiac tamponade was the true cause of death.

### 2.2. Case 2

**Figure 2.**
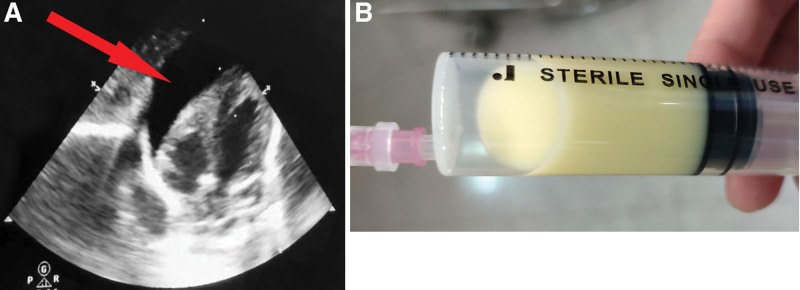
Echocardiogram and appearance of pericardial effusion in case 2. (A) An area of viscous and turbid fluid detected by echocardiography (red arrow); (B) Pericardial effusion appeared chylomicrons.

A male neonate, with BW of 1960 g, was born by cesarean at GA of 32^5/7^ weeks due to maternal PROM for 3 days. He appeared dyspnea and hypotonia, and pulmonary infection was revealed by CXR and blood examination (WBC 5.93 × 10^9^/L, NEU 2.88 × 10^9^/L, hemoglobin 142g/L, c-reactive protein [CRP] 15.64 mg/L). Noninvasive MV was continuously used after birth. Infection was effectively controlled by antibiotics treatment as evidenced by a decreased level of c-reactive protein (c-reactive protein 7.4 mg/L). Patent foramen ovale and PDA with a width of 3mm were found by echocardiography at PND 2. PICC was inserted through the left great saphenous vein at PND 2, and then PN was administrated via PICC every day. PICC tip positioning by postinsertion CXR were shown in Figure 1C and D. At PND 5 or 3 days after PICC placement, SpO_2_ declined to 80% and HR dropped to 125 beats/min. Immediate airway suctioning was performed, but no secretions were drained. Then a T-piece resuscitator was connected for positive ventilation with normal chest expansion and rebound; however, the SpO_2_ level further declined to 60% and HR dropped to 55 beats/min. Endotracheal intubation was promptly performed, and the baby was resuscitated with positive-pressure ventilation using a T-piece resuscitator in conjunction with chest compressions. Upon auscultation of the chest, bilateral lung sounds were found to be symmetrical and clear while heart sounds were faint, muffled and distant. An emergency echocardiography revealed a buildup of pericardial effusions (Fig. 2A), prompting immediate ultrasound-guided pericardiocentesis, which resulted in the drainage of 17 mL of chylopericardial effusion (Fig. 2B). Epinephrine, saline boluses and sodium bicarbonate were administered intravenously; however, there was no improvement in SpO_2_ or HR. The laboratory findings of pericardial effusion were as following: the routine test indicated that the pericardial fluid was turbid, viscous, appeared chylomicrons and contained a few red blood cells (2–4/HP) and leukocytes (3–6/HP), without any pus cells or coagulation; and biochemical test revealed a significant increase in triglyceride (17.9 mmol/L) and glucose levels (92.21 mmol/L) in the pericardial fluid, and the physicochemical properties of the fluid were consistent with the characteristics of PN in the PICC line, indicating that PN solution had entered into the pericardium.

## 3. Discussion

As shown in Table 1, from the beginning of the 21^st^ century, including our 2 cases reported herein, a total of 35 cases of PICC-related neonatal pericardial effusion/cardiac tamponade have been reported in China and abroad,^[3,4,6–18]^ with an average GA of (29.7 ± 3.7) weeks and an average BW of (1243.3 ± 517.3) g. Normally, there are about 5 ml of pericardial fluid in the pericardial cavity of newborns, and a small amount of fluid serves as lubrication to reduce friction during cardiac contraction.^[8]^ However, when a significant accumulation of fluid occurs within the pericardium, the pressure within the pericardial cavity increases, thereby limiting cardiac diastolic function correspondingly. The incomplete filling of the atria and ventricles leads to a reduction in cardiac output, resulting in severe hemodynamic disturbances, circulatory failure, and organ dysfunction.^[4,7,8]^ Therefore, vigilance is imperative in detecting the possibility of pericardial effusion when a neonate presents with poor responsiveness, pallor and/or cyanosis, altered respiratory rate, diminished heart sounds, tachycardia/bradycardia, weak peripheral pulses or prolonged capillary refill time,^[19,20]^ and early recognition and prompt management are paramount.

**Table 1:** Summary of reported cases of peripherally inserted central catheter (PICC)-related pericardial effusion/cardiac tamponade in neonates from 2000 to 2023.

Literature	Number of cases	Gestational age	Birth weight	PICC puncture site	PICC catheter tip position after pericardial effusion/cardiac tamponade	Treatment	Outcomes
Present literature	2	32^6/7^ wk, 32^5/7^ wk	1780 g, 1960 g	Left great saphenous vein for both cases	The right side of T 9 (1 case), traversed the vertebral column and terminated to the left of T 5 (1 case)	Conservative treatment (1 case), pericardiocentesis (1 case)	Dead (2 cases)
Nadroo et al^[3]^	2	34 wk, 26 wk	Not specified (1 case), 610 g (1 case)	Not specified (1 case), right anterior elbow vein (1 case)	Right atrium (1 case), unspecified (1 case), catheter migration (1 case)	Conservative treatment (2 cases)	Dead (2 cases)
Atmawidjaja et al^[4]^	1	33 wk	1360 g	Right antecubital fossa	Within the pericardial cavity	Pericardiocentesis	Dead
Pizzuti et al^[6]^	1	25 wk	620 g	Right basilic vein	Inferior CAJ	Pericardiocentesis	Cured
Khoo et al^[7]^	3	(30.3 ± 0.5) wk	(1256 ± 263) g	Right basilic vein (1 case), left antecubital fossa (1 case), right antecubital fossa (1 case)	CAJ (2 cases), right atrium (1 case), catheter migration (1 case)	Pericardiocentesis (3 cases)	Cured (3 cases)
Shannon^[8]^	1	27^+1^ wk	840 g	Left brachiocephalic vein	Right atrium	Pericardiocentesis	Cured
Zhang et al^[9]^	7	(30.9 ± 1.3) wk	(1307 ± 276) g	Right basilic vein (4 cases), Left basilic vein (2 cases), Right axillary vein (1 case)	Superior CAJ (1 case), right atrium (3 cases), left atrium (1 case), unspecified location (2 cases), catheter migration (5 cases)	Pericardiocentesis (3 cases), conservative treatment (4 cases)	Cured (5 cases), dead (2 cases)
Hou et al^[10]^	1	30^+1^ wk	1370 g	Right basilic vein	Superior vena cava (catheter tip bending)	Pericardiocentesis	Cured
Li et al^[11]^	1	33^+4^ wk	1550 g	Not specified	Right atrium	Pericardiocentesis	Cured
Warren et al^[12]^	2	22 wk, 41 wk	580 g, 3142 g	Not specified	Right atrium (2 cases)	Pericardiocentesis (1 case), conservative treatment (1 case)	Dead (2 cases)
Pezzati et al^[13]^	5	(27.4 ± 2.6) wk	(956 ± 333) g	Not specified	Right atrium (5 cases), catheter migration (5 cases)	Pericardiocentesis (3 cases), conservative treatment (2 cases)	Cured (3 cases), dead (2 cases)
Darling et al^[14]^	5	(27.6 ± 3.2) wk	(1182 ± 467) g	Upper limb (3 cases, detailed location not specified), lower limb (2 cases, detailed location not specified)	Right atrium (5 cases), catheter bending or angulation (5 cases)	Pericardiocentesis (2 cases), conservative treatment (3 cases)	cured (2 cases), dead (3 cases)
Haass et al^[15]^	1	25 wk	630 g	Right basilic vein	Not specified	Pericardiocentesis	Cured
Zou et al^[16]^	1	32^+1^ wk	1460 g	Median cubital vein	Right pulmonary artery	Pericardiotomy	Cured
Zarkesh et al^[17]^	1	30 wk	1190 g	Right upper limb (detailed location not specified)	Not specified	Pericardiocentesis	Cured
Cui et al^[18]^	1	32 wk	1570 g	Right basilic vein	Right atrium	Pericardiocentesis	Cured

PICC = peripherally inserted central catheter.

### 3.1. Etiology of pericardial effusion

All imaging data prior to the onset of both case 1 and case 2 did not reveal any evidence of pericardial effusion, thus primary pericardial effusion could be ruled out. The pathogenesis of secondary pericardial effusion could be classified into infectious and noninfectious etiologies. The former is typically caused by bacterial or viral infection, and the latter is often associated with autoimmune disorders, physical trauma, or neoplastic processes.^[21]^ Although both cases reported in this study had PROM and severe infection, the infection had been controlled after anti-infection treatment. In case 1, PICC induced pericardial effusion was confirmed to be chyle pericardial effusion by autopsy. In case 2, laboratory analyses suggested the composition of the pericardial effusion was consistent with PN, thus excluding infectious pericardial effusion. In addition, the absence of any definite deformity in both cases on cardiac ultrasound suggests that congenital heart disease is an unlikely cause of pericardial effusion. In our study, both cases received PICC placement prior to pericardial effusion, and no abnormalities in liver and kidney function, coagulation function, or myocardial enzymes were found. Therefore, iatrogenic factors after PICC insertion may be related to the occurrence of pericardial effusion. Other factors, such as MV and PDA were also existed in both patients, but there is no evidence to suggest their involvement in occurrence of pericardial effusion.

### 3.2. Influencing factors of PICC-related pericardial effusion

It has been demonstrated that the determinants of PICC-related pericardial effusion encompass gestational age, body weight, catheter insertion site, catheter tip position, catheter bending, angulation, catheter migration, and hypertonic fluid infusion, etc.^[10,22]^

**Figure 3.**
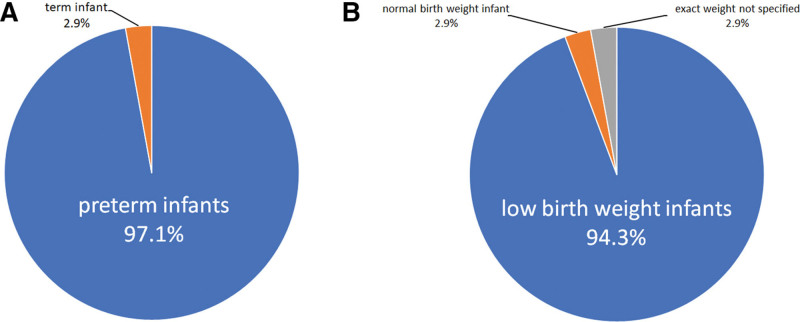
General information of 35 neonates with peripherally inserted central catheter (PICC)-related pericardial effusion/pericardial tamponade. (A) 34 cases (97.1%) were observed in preterm infants, while only 1 case (2.9%) was term infant. (B) There were 33 cases (94.3%) of low birth weight (<2500 g) infants, 1 case (2.9%) of normal birth weight and 1 case (2.9%) had unreported birth weight.

A descriptive statistical analysis found preterm infants (GA < 37 weeks)/low birth weight (BW < 2500 g) infants were accounted for the majority of the 35 cases reported (Fig. 3). The reasons are as follows: The current method for evaluating catheter length is inadequate in guiding the position of the PICC catheter tips in premature infants,^[9,23]^ yet proper placement of the catheter tip is essential to ensure the safety of PICC; There are numerous risk factors associated with catheter tip malposition in premature infants, including weight loss, changes in abdominal circumference, umbilical cord shrinkage and contracture, limb movement, and shortness of the superior vena cava;^[9,11,15]^ Premature neonates with immature cardiac tissue may exhibit myocardial weakness, which can be easily damaged by repeated friction between the catheter tip and heart wall during diastole and contraction. This damage can lead to endothelial cell injury, allowing fluid to diffuse into the pericardial cavity and form effusion.^[14,19,24]^

**Figure 4.**
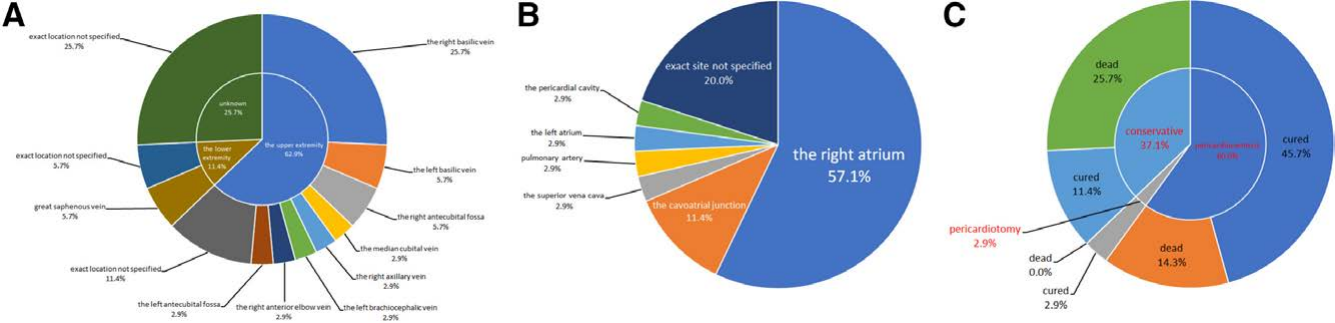
Peripherally inserted central catheter (PICC) placement, treatment and outcomes of 35 neonates. (A) Distribution of puncture sites: a total of 22 cases (62.9%) of PICC catheters were inserted via the upper extremity, with 9 cases (25.7%) from the right basilic vein, 2 cases (5.7%) from the left basilic vein and 2 cases (5.7%) from the right antecubital fossa, 1 case (2.9%) from the median cubital vein, the right axillary vein, the left brachiocephalic vein, the right anterior elbow vein, and the left antecubital fossa, respectively, and other 4 cases (11.4%) did not indicate the specific location in the upper extremity. A total of 4 cases (11.4%) were catheterized via the lower extremity, 2 cases (5.7%) were inserted from the great saphenous vein and 2 cases (5.7%) did not specify the exact location of the lower limb. Additionally, 9 cases (25.7%) did not provide any information about the location of PICC catheterization. (B) Position of PICC catheter tips: approximately 90% of PICC catheter tips were located within or in close proximity to the heart when PICC-related pericardial effusion/cardiac tamponade occurred, with 20 cases (57.1%) were located in the right atrium, 4 cases (11.4%) located at the CAJ, 1 case (2.9%) located in the superior vena cava, 1 case (2.9%) located in the pulmonary artery, 1 case (2.9%) located in the left atrium, and 1 case (2.9%) located in the pericardial cavity, while exact location was not specified for the remaining 7 cases (20.0%). (C) Treatment and outcomes: 21 cases (60.0%) underwent pericardiocentesis, 16 cases (45.7%) were cured after puncture, 1 case (2.9%) underwent pericardiotomy and also cured. In contrast, a total of 13 cases (37.1%) were treated conservatively (received no pericardiocentesis), and 4 cases (11.4%) were cured after conservative treatment.

There is currently no consensus on the optimal site for neonatal PICC insertion. Figure 4A illustrated that among the 35 neonates, the majority of PICC catheterizations were performed via the upper extremity. However, most PICCs were inserted through the lower extremities in our NICU, and both cases reported herein utilized the great saphenous vein. Qing et al discovered that neonates who underwent PICC insertion via the lower extremity exhibited a higher rate of first-attempt success, reduced incidence of catheter malposition, and fewer complications related to catheterization.^[25]^ The explanations are likely as follows: The upper extremity veins are relatively smaller and less readily exposed, whereas the lower extremity veins are clearly visible and exhibit minimal tortuousity, thereby enabling more precise catheter placement^[25,26]^; There are numerous venous branches in the upper extremity, and penetrating through multilayered deep fascia and muscle tissue poses a challenge^[27]^; The veins in upper extremity are typically oriented at an oblique angle, which may result in vascular injury during catheterization.^[26]^ Since the vein in the lower extremity is easier to puncture, the first-attempt success rate is higher, and the indwelling time is longer,^[28]^ it may be considered as a superior option for PICC placement. Specifically, the great saphenous vein is recommended as the primary choice, followed by the femoral vein.^[27]^

The optimal location of the PICC catheter tip remains controversial. Most studies suggested that the ideal location should be at the cavoatrial junction ^[1,7]^, rather than the right atrium.^[7,11,19,24]^ However, it should be noticed that risks still existed even when the catheter tip is at the junction, as it may potentially migrate into the right atrium.^[24,29]^ Most studies have shown that the catheter tip should be positioned within the vena cava and outside the cardiac silhouette, that is, at approximately 1 cm beyond the cardiac silhouette for premature infants and 2 cm beyond for full-term infants.^[7,8,10,11]^ No bending of the PICC catheter was observed on chest X-ray in case 1, however, 2 consecutive chest X-rays revealed drifting of the PICC catheter tip (Fig. 1A and B). In case 2, PICC catheter bending was evident prior to pericardial effusion (Fig. 1C and D). Therefore, radiographic imaging suggests a high risk of catheter tip malposition in both cases. It was also confirmed by chest radiography that most of PICC catheter tips were located within or in close proximity to the heart when symptoms of pericardial effusion/cardiac tamponade were present (Fig. 4B). Among them, 13 cases (37.1%) exhibited catheter migration, while 7 cases (20.0%) demonstrated catheter bending or angulation. Studies have indicated that the primary mechanisms of PICC-induced pericardial effusion/cardiac tamponade are mechanical damage caused by catheter tip and chemical stimulation due to hypertonic fluid on the atrial wall.^[8]^ When the catheter tip is located in the right atrium, it comes into contact with and repeatedly rubs against the atrial wall, leading to endocardial inflammation and necrosis.^[12]^ This subsequently progresses to myocardial injury and perforation, ultimately resulting in pericardial effusion/cardiac tamponade.^[10,24]^ Catheter migration is another high-risk factor for PICC-related pericardial effusion. Nadroo et al found that soft tissue around the catheter would be squeezed during adduction of the shoulder joint or flexion of the elbow, and the venous catheter would displace from the heart; whereas concurrent shoulder joint adduction and elbow flexion would result in its retraction towards the heart.^[3]^ Therefore, maintaining arm adduction and elbow flexion during X-ray positioning can effectively reduce the risk of pericardial effusion caused by PICC displacement into the right atrium due to arm movement,^[10]^ and the exposed end of the PICC should be securely affixed with adhesive tape to minimize catheter migration.^[4,9]^ To ensure accurate positioning of the catheter tip, X-ray or cardiovascular ultrasonography should be performed at least twice a week. However, the sensitivity and specificity of X-ray localization are inferior to those of cardiovascular ultrasonography. Moreover, due to the similar imaging density between pericardial effusion and the heart itself on chest X-ray, it is challenging to distinguish them. Therefore, cardiovascular ultrasonography is the preferred method for positioning PICC tip and diagnosing pericardial effusion/cardiac tamponade.^[7,8,11]^ Unfortunately, even with correct catheter positioning, the occurrence of pericardial effusion/cardiac tamponade cannot be completely prevented.^[15]^ The perforation may result from repeated rubbing of the catheter tip against the inner wall of the pericardial vena cava with each heartbeat or continuous infusion of hypertonic PN, leading to transmural injury and necrosis that ultimately results in pericardial effusion.^[9–11,19]^ Both cases in this study received PN infusion through PICC prior to the onset of pericardial effusion/cardiac tamponade. In case 1 and case 2, the highest concentration of triglyceride in PN reached 4.9 % and 4.5 % respectively, and the triglyceride infusion rate reached 0.18g/kg and 0.11g/kg per hour respectively. The triglyceride infusion rates in both cases were close to or even exceeded the upper limit recommended by guidelines (0.15 g/kg per hour).

### 3.3. Outcomes in neonates receiving different interventions

Prompt intervention is necessary upon diagnosis of pericardial effusion/cardiac tamponade. Zhang et al suggested that conservative treatment such as fluid restriction and diuresis, may be appropriate for mild to moderate pericardial effusions with hemodynamic stability.^[9]^ There were also reports of successful rescue by either repositioning the catheter tip away from the right atrium or direct drainage of the effusion via PICC.^[10]^ However, once echocardiography reveals cardiac chamber compression and deformation, restricted diastole and systole, and reduced cardiac output, prompt pericardiocentesis is necessary. The subxiphoid percutaneous approach is a safer and more reliable option.^[6]^ As shown in Figure 4C, retrospective analysis of 35 cases also demonstrated a significantly higher survival rate among patients who underwent pericardiocentesis or pericardiotomy (17/22 or 77.3%) compared to those who received conservative treatment (4/13 or 30.8%).

As described above, not all babies could be successfully cured through pericardiocentesis, nevertheless, the failure to promptly recognize pericardial effusion/cardiac tamponade due to atypical manifestations and the absence of timely pericardiocentesis in case 1 represent potential limitations associated with this case report. These limitations do not impact the findings and conclusions of this study; instead, they emphasize the significance of vigilance towards the possibility of pericardial effusion/cardiac tamponade in neonates with PICCs who suddenly present with unexplained hemodynamic antecedents, and prompt response as well.

## 4. Conclusion

Considering the high mortality rate of PICC-related pericardial effusion/cardiac tamponade and the frequent misdiagnosis due to lack of specific manifestations, this study summarizes experiences and lessons learned to provide guidance for early warning, timely recognition and treatment of pericardial effusion/cardiac tamponade in order to save infants lives. Combined with the present cases and relevant literature, conclusions were summarized as follows: Recognize the subpopulation of neonates at high risk for the development of pericardial effusion/cardiac tamponade following the PICC catheterization such as immaturity/low birth weight infants, malposition of catheter tip, catheter bending, catheter angulation, and catheter migration. PICC should only be used when deemed necessary and promptly removed if no longer required. Keeping the head and neck in alignment, ensuring appropriate arm adduction and elbow flexion during X-ray positioning procedures. The preferred site for PICC puncture is the great saphenous vein or other veins in the lower extremities. The catheter tip should be positioned within the vena cava, maintaining a distance of 1 to 2 cm from the heart. Properly securing the exposed end of the PICC is essential to prevent catheter dislodgement. When respiratory and circulatory instability occurs, an emergency cardiovascular ultrasound examination should be promptly conducted to confirm the presence of pericardial effusion/cardiac tamponade; meanwhile the infusion must be ceased immediately and cannot be resumed until normalcy is confirmed. In the event of a diagnosis of pericardial effusion/cardiac tamponade, immediate ultrasound-guided pericardiocentesis should be performed alongside routine cardiopulmonary resuscitation. The puncture fluid should then be submitted for examination to determine its physicochemical properties and identify the source of effusion. It is possible to try to extract pericardial effusion via PICC; however, timely pericardiocentesis should still be performed in cases of severe symptoms in children. PN formula should strictly control the concentration, osmotic pressure, and infusion rate, with particular attention to the concentration of fat emulsion and infusion speed. In case where a child died from cardiac tamponade, it may be advisable to suggest an autopsy to investigate the presence or absence of congenital structural abnormalities in the lymphatic system. For instance, malformations of the thoracic duct or the expansion and rupture of the thoracic duct caused by partial obstruction stenosis leading to chylous leakage, and abnormal channels between the lymphatic duct and the pericardial cavity. Improve the skills of pericardiocentesis for neonatologists. The informed consent of family members should be obtained prior to PICC catheterization, and they should be informed about the potential risks of complications such as pericardial effusion/cardiac tamponade. All these measures aim to improve the refined management of neonatal PICC catheterization, establish an early warning/disposal process mechanism, reduce the incidence of complications related to PICC insertion such as pericardial effusion/cardiac tamponade, and ultimately improve children prognosis.

## Acknowledgments

We are most grateful for the information provided by Medical Records and Statistics Room of Sichuan Provincial People Hospital.

## Author contributions

**Conceptualization:** Binzhi Tang.

**Data curation:** Yucen Liu.

**Investigation:** Yucen Liu, Binzhi Tang.

**Methodology:** Maojun Li, Wei Shi, Binzhi Tang.

**Software:** Yucen Liu, Binzhi Tang.

**Writing – original draft:** Yucen Liu, Binzhi Tang.

**Writing – review & editing:** Maojun Li, Wei Shi, Binzhi Tang.
